# TLRs in Hepatic Cellular Crosstalk

**DOI:** 10.1155/2010/618260

**Published:** 2010-08-30

**Authors:** Amelie E. Bigorgne, Ian Nicholas Crispe

**Affiliations:** ^1^Malaria Program, Seattle BioMed, 307 Westlake Avenue North, Suite 500, Seattle, WA 98109, USA; ^2^Department of Immunology, University of Washington, 1959 NE Pacific Street, Seattle, WA 98195, USA

## Abstract

Toll-like receptors (TLRs) are expressed on all major subsets of liver cells. Both exogenous ligands derived from pathogens, and endogenous ligands that are products of cellular injury, engage these receptors and activate aspects of innate immunity. These receptors play a role in viral and parasitic infections of the liver, in ischemia-reperfusion injury, and in toxic liver damage, promoting antipathogen immunity but also hepatocellular injury and fibrogenesis. However, TLRs may also participate in negative feedback that limits tissue injury. In the complex environment of the liver, TLRs participate in pathologic cascades involving multiple cell types, manifesting their effects both through cell-autonomous actions, and via cellular crosstalk. In this paper we survey the involvement of TLRs in these diverse processes.

## 1. Introduction

The Toll-like receptors (TLRs) form a multigene family that is well conserved between human and murine species. These receptors are cell surface or intracellular receptors for molecular signatures characteristic of viruses, bacteria, and parasites, including features of their nucleic acids, proteins, and lipid and carbohydrate components. The pathogen-associated molecular patterns (PAMPs) engaged by TLRs are basic features of these microorganisms that cannot readily be modified by genetic mutation, thus that are features of entire categories of microorganisms. A prime example is the lipopolysaccharide (LPS) endotoxin of the cell walls of Gram-negative bacteria, which engages a cell surface member of the TLR family, TLR4, activating multiple downstream signaling pathways that result in the synthesis of cytokines and interferons. TLRs share functional similarities, and downstream effector mechanisms, with other pathogen recognition systems such as the RIG-I like proteins that detect viral nucleic acids, and the NOD-like receptors that respond to bacterial cell wall elements.

All of the known TLRs are expressed in the liver, and this is likely to be biologically important since the liver receives blood from the intestine, which is an internal body surface exposed to PAMPs derived from harmless commensal bacteria in the gut lumen as well as potentially antigenic components of the diet and from time to time, invasive microorganisms.

Hepatic injury is associated with an increase of liver exposure to bacterial products, but the healthy liver is able to develop a tolerance towards bacterial products coming from the gut. Specifically, the exposure of liver sinusoidal endothelial cells (LSECs) to low levels of LPS results in the loss of their TLR4 expression, resulting in LPS insensitivity [1]. This effect is not limited to homologous ligand, since the administration of the TLR3 ligand, poly I : C, also downregulates LPS sensitivity on Kupffer cells (KCs) [2]. In hepatocytes, this mechanism depends on SOCS-1 which interacts with TIRAP in the TLR signaling pathway [3].

In the liver, immune responses are complicated by the immune competence of many populations of cells, including an unusual assembly of lymphocytes in which Natural Killer (NK) cells and CD8+ T cells are unusually abundant, as well as Dendritic Cells (DCs), KCs, LSECs, hepatic stellate cells (HSCs), hepatocytes, and bile duct cells. Any or all of these cell types may respond to TLR signals, and any of them may act as antigen-presenting cells (APCs) that can engage T cells. Inflammatory or immune pathologies that converge on the hepatocyte (such as hepatocellular injury and regeneration), or the HSC (fibrosis, cirrhosis), very likely involve other cell types. For example, innate immune signals may activate KCs, the KCs may elaborate cytokines, and these cytokines may act on HSCs, either promoting or suppressing fibrogenesis. Here we address the issue of how TLRs may be involved in such cellular cross-talk in liver immunopathology. 

The analysis would be more straightforward if each liver cell type expressed a characteristic set of TLRs. However, there is very little segregation of TLR expression: studies with both purified cell cultures and cell lines support the idea that all liver cell populations express essentially all TLRs at the mRNA level. Comprehensive studies of the responsiveness of individual cell types to a full range of TLR ligands are few. At present, no specific liver cell population can be identified as central in TLR-mediated pathologies. Furthermore, the effects of TLR ligation vary from cell to cell. 

While TLRs can initiate innate immune cascades through the recognition of exogenous PAMPs, they also recognize endogenous signals released by damaged cells. Thus, dying cells release RNA, which can engage TLR3; nuclear DNA that can engage TLR9; and HMBG1 (high mobility group box protein 1) that can engage TLR4. This gives endogenous injury signals, like exogenous PAMPs, access to both the MyD88 and the TRIF signaling pathways. This raises the possibility that immunopathology, initiated by a TLR-mediated response to a PAMP, can result in tissue damage that sustains itself through other TLR-mediated mechanisms. In the liver, TLRs are involved in infections (HCV, HBV), but also in “sterile injury” models including Concanavalin A (ConA)-induced hepatitis, bile duct ligation, partial hepatectomy, acetaminophen toxicity, and ischemia-reperfusion injury. TLRs and particularly TLR4 play a key role in liver regeneration, in alcoholic liver injury, in the development of steatosis, and in the recruitment of activated CD8^+^T cells to the hepatic sinusoids [4]. In addition, tissue culture models reveal some pathways of cell-cell interaction triggered by TLR engagement. In our own experiments, TLR2, TLR3, and TLR4 activation of human Kupffer cells resulted in differential secretion of IL-18 and IL-10, which coordinately regulated the activation and IFN-*γ* synthesis of cocultured human liver NK cells [5].

Downstream effects of TLR signaling in diverse models include hepatocellular injury, measured most often as an increase in serum alanine aminotransaminase (ALT); fibrogenesis, measured by the upregulation of timp-1, collagen, and *α*-smooth muscle actin expression; steatosis quantified mainly by hematoxylin eosin saffron (HES) histochemical staining of lipid droplets; liver regeneration assessed *via* hepatocyte mitotic activity and the restoration of liver mass; and anti-pathogen effects such as suppression of HCV replication or malaria parasite load. The detailed articulation of these numerous interacting pathways is still some way in the future. We can, however, explore some structuring ideas through the current literature, and try to discern some common themes in TLR involvement in liver pathology.

## 2. TLRs Responding to Endogenous Ligands

While it is clear that TLRs may respond to molecules released by injured cells, there are also experimental models induced by mechanical or toxic stresses that do involve *bona fide *bacterial products, and these cases need to be distinguished from the involvement of TLRs in “sterile injury” where the ligands are truly endogenous. Bacterial endotoxemia contributes directly to liver injury in apparent unrelated hepatic pathologies such as cholestasis, chronic alcoholic hepatitis, or steatohepatitis. In bile duct-ligated animals, pretreatment with intraperitoneal LPS upregulates the expression of TLR4 and MyD88 [6], as well as CD14 and CD68 [7], and confers hypersensitivity to LPS in fibrotic livers. The increase in proinflammatory cytokines (TNF-*α* and IL-6) secretion was observed as early as two hours after LPS administration in BDL rats. These models of liver injury in apparently unrelated hepatic pathologies.

In other models, TLRs are involved but the source of their ligands is less clear. For example, after hemorrhagic shock, endogenous damage-associated molecular patterns (DAMPs) are released from cells during traumatic injury allowing them to interact with TLRs. In particular, TLR4 expression is required on bone marrow-derived cells and on liver parenchymal cells to detect systemic and remote organ response to hemorrhagic shock [8]. However, it is unclear if this model also results in LPS translocation across the gut.

In nonalcoholic steatohepatitis, a direct link exists between TLR4 and Kupffer cells in the pathogenesis of liver injury. In a methionine choline deficiency (MCD) diet-induced model of steatohepatitis, histological evidence of liver inflammation, portal endotoxemia and enhanced TLR-4 expression occurs in wild-type mice fed the MCD diet. In contrast, injury and lipid accumulation markers were significantly lower in TLR4 mutant mice. Targeted destruction of Kupffer cells with clodronate liposomes blunted histological evidence of steatohepatitis and prevented increases in TLR4 expression, induced increases in the production of TNF-*α*, ICAM-1, and significantly impaired the development of hepatic injury. In support of the idea that the expression of TLR4 on Kupffer cells is essential for injury, destruction of these cells also prevented increases in TLR-4 expression [9].

In alcoholic liver disease, low expression of the anti-inflammatory factor GILZ (Glucocorticoid Induced Leucin Zipper) in monocytes contributes to liver inflammation and hypersensitization to LPS. GILZ messenger RNA (mRNA) levels were lower in the livers of patients with AH versus those without AH. A treatment with glucocorticoids enhances GILZ expression and abrogates macrophage sensitivity to LPS and subsequent proinflammatory cytokine secretion [10].

In this model, TLR4 is required for liver injury but MyD88, its principal downstream effector, is not necessary for injury. Alcohol feeding results in a significant increase in liver injury in wild-type (wt), TLR2−/−, and MyD88−/− but not in TLR4−/− mice [11]. The expression of inflammatory mediators (TNF-*α* and IL-6) and the TLR4 coreceptors (CD14 and MD2) significantly increases in livers of alcohol-fed wt, TLR2−/−, or MyD88−/−, but not in TLR4−/− mice, compared to controls. Alcohol feeding also induces nuclear factor-kappaB activation in a TLR4-dependent, but MyD88-independent manner. This shows that while TLR4 deficiency was protective, MyD88 deficiency failed to prevent alcohol-induced liver damage and inflammation by implication suggesting that the alternative TLR4 signaling pathway involving the adapter protein TRIF, and also IRF-7, was involved.

A very recent report shows that inflammation occurs after both major trauma and infection injury [12]. Mitochondrial DAMPs (MTDs) include formyl peptides and mitochondrial DNA. Mitochondrial DNA activates human polymorphonuclear neutrophils (PMNs) through TLR9. The assessment of MMP8 protein expression—a marker of neutrophil infiltration—was increased in whole livers of rats injected intravenously with mitochondrial DAMPs whereas control rats showed no evidence of hepatic inflammation. 

After tissue trauma, mitochondrial DAMPs that express at least the two molecular signatures, formyl peptides, and mtDNA, act on pattern recognition receptors recognizing bacterial PAMPs. These activate PMN in the circulation rather than at specific targets, inciting nonspecific attack on multiple organs, including the liver, while suppressing chemotactic responses to infective stimuli. 

Other intracellular “alarmins” may similarly be important after injury, and other immune cells probably respond to MDTs. Injury-derived MDTs, however, are clearly recognized by innate immunity using pattern recognition receptors that alternatively sense bacteria. This novel model may explain why responses to these ancient “enemies within” released by injury can mimic sepsis.

## 3. TLRs May Mediate Injury-Limiting as well as Injury-Promoting Pathways

One good example of this “injury limiting effect” is the response of conventional DCs to TLR9 ligation by DNA released from cells during ischemia-reperfusion injury. By secreting IL-10, these DCs respond to damage-associated patterns released by injured cells, providing the host with protection from progressive damage, potentially limiting tissue injury in the presence of dying cells [13].

Similarly, the ligation of TLR9 inhibits NF-*κ*B binding activity in T cells, and increases survival of mice in a model of ConA-induced hepatitis. Liver injury—as measured by circulating ALT levels—decreases after pretreatment with CpG oligodeoxynucleotides sequences that can engage TLR9 [12]. However this is not a property of TLR9 in general, since this receptor promotes liver injury in acetaminophen injury [14], in ischemia/reperfusion [13], and in nonalcoholic steatohepatitis [15].

In acetaminophen injury, acetaminophen treatment results in hepatocyte death and the free DNA released from apoptotic hepatocytes activates TLR9. This triggers a signaling cascade that increases transcription of the genes encoding pro-IL-1*β* and pro-IL-18 in LSECs. TLR9 antagonists and aspirin reduced mortality from acetaminophen hepatotoxicity [16]. In ischemia/reperfusion injury, TLR4, but not TLR2, is specifically required to initiate the tissue-damaging cascade, as manifested by liver function (serum ALT levels), pathology, and local induction of proinflammatory cytokines/chemokines (TNF-*α*, IL-6, and CXCL10) [14].

In a model of liver injury induced with acetaminophen, there was MyD88-dependent recruitment of neutrophils into the liver, as assayed by the abundance of the neutrophil enzyme myeloperoxidase (MPO) [17]. This was due not to TLR signaling, but required the IL-1R expressed on non-bone marrow-derived cells. Sterile neutrophilic inflammation is thought to contribute to the pathogenesis of acute ischemia-induced liver injury and to impair healing. Blocking such sterile inflammation is a potentially attractive strategy to limit the damage of acute sterile inflammation and to stop the ongoing damage in chronic inflammation from progressing to liver injury. 

In a model of segmental liver ischemia-reperfusion injury, the treatment of wt mice with an inhibitory cytosine-guanosine dinucleotide (iCpG) sequence reduced significantly the serum ALT and inflammatory cytokines after liver ischemia-reperfusion injury, and the same was seen in TLR9-deficient mice. Liver damage was mediated by bone marrow-derived cells because wt mice transplanted with TLR9−/− bone marrow were protected from injury. Injury in wt mice partly depends on TLR9 signaling in neutrophils, which enhanced production of ROS, IL-6, and TNF-*α*. *In vitro*, DNA released from necrotic hepatocytes increased cytokine secretion in liver nonparenchymal cells and neutrophils through a TLR9-dependent mechanism. Inhibition of both TLR9 and HMGB1 caused maximal inflammatory cytokine suppression in neutrophil cultures and conferred even greater protection from ischemia-reperfusion injury *in vivo* [13]. 

In diet-induced obesity, TLR9−/− mice show less steatohepatitis and liver fibrosis than wt mice. Among inflammatory cytokines, IL-1*β* production is suppressed in TLR9−/− mice. Kupffer cells produce IL-1*β* in response to CpG oligodeoxynucleotides leading to steatosis and inflammation [15]. Similarly, CpG DNA promotes liver injury in the presence of D-Gal, promoting apoptotic death in hepatocytes [18]. Taken together, these data do not yield a simple model for the involvement of TLR9 signaling in modulating liver injury. The most straightforward interpretation is that TLR9 has both pro- and anti-injury effects, depending on the cell types concerned and their interactions. 

Two single nucleotide polymorphisms (SNPs) of the TLR4 gene emerge as conferring protection from fibrosis progression compared to wt. The study of the functional linkage of this SNP to HSCs responses show that both HSCs from TLR4−/− mice and a human HSC line reconstituted with either TLR4 D299G and/or T399I cDNAs were hyporesponsive to LPS stimulation compared to those expressing wt TLR4, as assessed by the expression and secretion of LPS-induced inflammatory and chemotactic cytokines (MCP-1 and IL-6). The conclusion is that TLR4 D299G and T399I SNPs that are associated with protection from hepatic fibrosis reduce TLR4-mediated inflammatory and fibrogenic signaling and lower the apoptotic threshold of activated HSCs. These findings provide a mechanistic link that explains how specific TLR4 SNPs may regulate the risk of fibrosis progression [19].

Conversely, when TLRs do not intervene in injury-limiting pathways, they can promote liver failure through direct interaction with mediators promoting injury. Although the receptor for advanced glycosylation end products (RAGEs) has been shown to interact with HMGB1, the recent identification of direct recognition of HGMB1 with different TLRs has confirmed the wide range of possible interactions. The HMGB proteins have been described to play a role as late mediators of lethality in sepsis as well as in cells undergoing necrosis, but not in cell death due to apoptosis nor from cells exposed to inflammatory cytokines. HMGB1 is a nuclear factor, which is released from injured cells, including hepatocytes. It is argued that HMGB1 interacts with TLR4, since TLR4 defective (C3H/HeJ) mice exhibits less damage in the hepatic ischemia-reperfusion model than wt C3H/OuJ mice [20].

TLR9 sits at the interface of microbial and sterile inflammation by detecting both bacterial and endogenous DNA. Released in the extracellular compartment during acute inflammatory responses, HMGB1 also interact with TLR2 [21] and TLR9 [22]. Unlike LPS, which primarily increased the activity of IKK-*β*, HMGB1 exposure resulted in activation of both IKK-*α* and IKK-*β*. Kinases and scaffolding proteins downstream of TLR2 and TLR4 were involved in the enhancement of NF-*κ*B-dependent transcription by HMGB1. Transfections with dominant negative constructs show that TLR2 and TLR4 were both involved in HMGB1-induced activation of NF-*κ*B. Interactions of HMGB1 with TLR2 and TLR4 may provide an explanation for the ability of HMGB1 to generate inflammatory responses that are similar to those initiated by LPS [21]. TLR3 may also act to modulate aspects of liver inflammation by activating NKT cells that can eliminate gamma-delta T cell through apoptosis [23], thus changing the cellular makeup of inflammatory infiltrates.

Stellate cells are subject to regulation both through their own TLRs and via cross-talk. Thus, stellate cells are maintained in an undifferentiated state by interaction with fresh LSEC acting via VEGF and NO [24]. 

As mammalian cells undergo apoptosis, genomic DNA undergoes significant modifications, which include caspase-mediated cleavage but also aberrant methylation and oxidative damage. Such changes may result in enrichment in CpG sequences in comparison to random DNA from the human genome. As a sensor for cell injury, HSCs phagocytose apoptotic hepatocyte bodies with subsequent regulation of TGF-*β* and collagen-*α*1 mRNA [25]. The consequence is TLR9-dependent HSC differentiation as well as a stop signal to retain HSC at sites of hepatocyte apoptosis [26]. Other groups have shown that TLR4 downstream activation can have important functional consequences on hepatic stellate cells. TLR4 activation in hepatic stellate cells sensitizes HSCs to TGF-*β*-induced signals and upregulates chemokine secretion and induces chemotaxis of Kupffer cells [27, 28]. In particular, LPS induces signal transduction and upregulates chemokines (IL-8, CCL2) and adhesion molecules (VCAM-1 and ICAM-1) in activated human HSCs from patients with hepatitis C virus induced cirrhosis [27].

## 4. TLRs in Host Defense against Hepatocellular Pathogens

TLRs were first identified in *Drosophila melanogaster* as a genetic element, the lack of which predisposed adult flies to lethal fungal infection [29]. Recognition of the significance of such nonrearranging receptors led Medzhitov and Janeway to search for homologous molecules in the mouse and human genomes, [30], while a convergent line of research using positional cloning identified the receptor for LPS as a TLR [31]. While additional roles of these molecules continue to emerge, the principal phenotypes of TLR-deficient mice are associated with increased susceptibility to infection. The TLRs also play this role in the liver. 

Viruses interacting with host cells can modulate expression and function of TLRs. In chronic hepatitis C virus (HCV) infected patients, expression of TLR4, 7 and 8 is increased in peripheral CD14^+^ cells together with circulating levels of TNF-*α*, IL-6, and IL-12p35. The incubation of PBMC with HCV core protein triggers the expression of TLR2 and suppresses TLR4 and TLR7 [32]. Activation of nonparenchymal cells such as KCs and LSECs with TLR ligands leads to the secretion of IFN-*β*, which powerfully suppresses HCV replication [33]. In the same way, while the expression of messenger RNA encoding all TLRs is detectable in HSCs, the spectrum of TLR ligands, which are capable to induce secretion of antiviral cytokines, is restricted to TLR3 in human HSCs. Moreover, such ligation results only in IFN-*β* sufficient to suppress the replication of either LCMV or HCV, but not in the synthesis of relevant amounts of other IFNs (i.e., neither IFN-*γ* nor IFN-*α*) [34].

These host defense-promoting effects of TLR engagement are not only antiviral. TLR2, 3, 4, and 9 ligands can reduce the liver load of parasites in murine *Plasmodium yoelii* infection. In particular, CpG as a TLR9 ligand causes an 88% decrease in hepatic parasite load, and in mice challenged with 100 sporozoites, results in complete suppression of parasitemia for at least 14 days [35]. This effect of CpG was accompanied by increases in hepatic IL-12 and TNF-*α* as well as IFN-*γ*, and decreases in IL-10 and TGF-*β*1. The effect of Kupffer cell depletion is to abrogate these effects and restore parasite load. It therefore seems likely that CpG was eliciting a three-way cross-talk among liver cells: the activation of Kupffer cells resulting in IL-12 and TNF-*α*; the activation of either NK cells or T cells since these are the main sources of IFN-*γ*; and hepatocytes, the cells in which the parasite develops or fails to develop under conditions stimulating TLR2, 3, 4 or particularly TLR9. *Plasmodium berghei* infection induces IL-12 through MyD88-dependent pathways, but not TLR2, 4, and 6 [36]. This secretion of IL-12 induces hepatocyte killing through hepatic CD1d-independent DX5+ T cells through a perforin-dependent mechanism [37]. Hepatic *Listeria monocytogenes *infection induces IL-12 and IL-18 production in Kupffer cells through TLR/MyD88 signaling, which stimulates NK cells to produce IFN-*γ*, and this is critical for eradication of *Listeria* organisms from the host [38, 39].

The evolutionary history of TLRs, and their role in directly binding to PAMPs, may suggest that the primary TLR function is to initiate the innate immune response to pathogens and to condition accessory cells in ways that promote the induction of adaptive immunity. However, TLR activation can also be a mechanism through which responses are sustained. One example comes from the response to an Adenovirus vector, in which both the innate and adaptive immune response depend on TLR2 and TLR9 [40]. The key point is that Adenovirus was able to activate signaling through ERK1/2 in Kupffer cells, but that such activation was independent of TLR2 and MyD88. However, the sustained activation of ERK1/2 required both TLR2 and MyD88. With respect to NF-kB activation, early activation required MyD88 but not TLR2, while sustained activation required both MyD88 and TLR2. Cytokine and chemokine responses also required MyD88 and TLR2. It is not entirely clear how this works, but the most likely model is that the innate immune responses, initiated by direct activation of ERK1/2, release endogenous ligands that signal via a receptor coupled to MyD88, and for full immune activation also act via TLR2. Such ligands have not yet been identified. 

A strikingly similar role for TLR signaling in sustaining an innate response was found in a very different experimental model. In poly-microbial septic peritonitis, in the absence of any exogenous viral stimulus, the initial cytokine and chemokine burst was increased in TLR3−/− mice; however this response was more rapidly curtailed, and these mice were protected from the lethal effects of sustained inflammation [41]. The same was found in an ischemic gut injury model. Furthermore, the investigators found that RNA released from apoptotic neutrophils could activate macrophages from wt, but not from TLR3−/− mice, suggesting that a positive feedback loop was acting through neutrophil recruitment and apoptosis, activation of macrophages by released RNA, and macrophage activation leading to sustained inflammation. 

Integrating these elements, we arrive at a picture where TLRs have two distinct roles in responses to exogenous infection. First, they may act directly as sensors of PAMPs synthesized by pathogens, and in this role they are likely to initiate the first steps in an immune response. But in addition, TLRs may amplify or sustain an immune response by signaling in response to exogenous molecular products of tissue damage, such as DNA (TLR9), HMGB1 (TLR4), and RNA (TLR3). In the early days of the recognition of the importance in immune activation of diverse signals apart from T and B cell receptor ligation, it is argued that lymphocytes are responding not to “nonself” molecules but to “danger”, which can only be construed as a metaphor for tissue injury [42]. Alternatively, it was counter-argued that such “danger” could be better understood as molecular signatures characteristic of pathogens, the signals that were subsequently named PAMPs [43]. Now, through an analysis of the diverse roles of TLRs in infectious disease in the liver and elsewhere, we can see that both positions were correct. What is more, “danger” and PAMPs activate and sustain inflammation through the same family of receptors.

## 5. TLR Signals Are Transmitted via Cell-Cell Crosstalk

Cross-talk is when a TLR acts on one cell but the biological effect is transmitted to a different cell type. Few examples of TLR-driven cross-talk have been explored in the liver, but the examples that exist implicate several major cell types in interactions of this kind.

We have studied the impact of TLR signaling in human Kupffer cells, obtained from fresh human liver lobes sampled during the process of living donor liver transplantation. This procedure yields intrahepatic leukocytes that are rich in human KC and human liver NK cells, and these were used in cross-talk experiments *ex vivo*. Engagement of TLR3 resulted in powerful NK cell activation in KC-NK cell cocultures, but not when the two cells were separated in a Transwell tissue culture system [5]. Stimulation via TLR2 or TLR4 resulted in less dramatic NK cell activation, but the full activation of NK cells was restored by blocking IL-10. Thus the TLR ligands that activated the MyD88 signaling pathway induce IL-10 along with proinflammatory cytokines, and inhibit NK cells; the TLR3 ligand, working only via the TRIF pathway, does not induce IL-10. Conversely, the main NK cell-activating signal is IL-18, synthesized in response to all three TLR ligands [5]. Thus, Kupffer cells are integrating signals from different TLRs, and integrating them to modulate NK cell activity via cross-talk.

While these experiments in human cells emphasize the effects of IL-18 on NK cells, we have also explored IL-18 action in murine NK-T cells. In these cells, also, it is a powerful activating factor [44]. Hepatitis C virus-encoded core protein is able to interfere with TLR-mediated activation of human Kupffer cells. Specifically, core binds to TLR2, and this can induce diverse proinflammatory cytokines [45, 46], but also IL-10. We also show that HCV core acting on human Kupffer cells can suppress other antiviral mechanisms, including type 1 IFN and the upregulation of TRAIL [24]. Thus, viruses can exploit TLRs to disable immunity.

One important negative regulatory effect of cross-talk is the recently discovered action of LSEC in suppressing the transdifferentiation of HSCs via nitric oxide [24]. The key point is that only resting LSECs have this effect, since endothelial cells harvested from a liver undergoing capillarization do not suppress HSC trans-differentiation. The LSECs are influenced in their capacity to make nitric oxide by VEGF, which therefore acts as an antifibrotic factor. While there is no evidence relating to the influence of TLRs in NO synthesis by LSECs, there is a strongly analogous case. In neurovascular endothelium, NO synthesis was induced by *Neisseria meningitidis,* and this effect was blocked by antibodies to TLR2 and TLR4—but not to TLR9 [47]. Extrapolating to the liver, where LSECs are constitutively exposed to LPS concentration ranging from 100 pg/mL to 1 ng/mL, it seems likely that such low level TLR4 engagement supports NO synthesis and maintains HSCs in their resting state. 

TLR4 signaling enhances TGF-*β*1 in HSCs by downregulating the TGF-*β*1 pseudo-receptor Bambi, thus stimulating fibrosis. Quiescent hepatic stellate cells are the predominant targets through which TLR4 ligands—but not TLR2 ligands—are required to promote fibrogenesis. In quiescent HSCs, TLR4 activation upregulates chemokine secretions induces chemotaxis of KCs, and allows for unrestricted activation by KCs [28]. 

We can therefore see signals that are initiated by TLRs participating in cross-talk between KCs and NK cells, and between KCs and HSCs, and we can envisage mechanisms through with TLRs modulate cross-talk between KCs and NK-T cells, and between LSECs and HSCs. Since the expression of TLRs is almost universal among liver cell subsets, the documentation of such TLR-driven cell-cell interactions is sure to increase.
